# Interleukin-6 on postoperative day three as an early predictor of infections following laparoscopic gastric cancer resection

**DOI:** 10.1186/s12893-024-02381-8

**Published:** 2024-03-19

**Authors:** Yongzhou Huang, Lei Yang, Wenchang Yang, Pei Zhou, Qi Jiang, Weizhen Liu, Yuping Yin, Xiangyu Zeng, Peng Zhang, Kaixiong Tao

**Affiliations:** 1 Department of Gastrointestinal Surgery, Union Hospital, Tongji Medical College, Huazhong University of Science and Technology, No. 1277 Jiefang Avenue, Wuhan, Hubei Province 430022 China; 2 Department of General Surgery, First Affiliated Hospital of Shihezi University, Shihezi, Xinjiang 832008 PR China

**Keywords:** Interleukin-6, Procalcitonin, C-reactive protein, Gastric cancer, Postoperative complications

## Abstract

**Background:**

To investigate the role of C-reactive protein (CRP), procalcitonin (PCT), and interleukin-6 (IL-6) as early predictors of infectious complications after laparoscopic gastric cancer surgery.

**Methods:**

Patients who underwent laparoscopic gastric cancer surgery between January 2020 and June 2022 were retrospectively enrolled. IL-6, PCT, and CRP levels were assessed before surgery and on postoperative days (PODs) 3 and 5. Differences in serum IL-6, PCT, and CRP levels between the infected and non-infected groups were compared. The diagnostic accuracy was determined using the area under the receiver operating characteristic curve (AUC).

**Results:**

A total of 206 patients were enrolled, and 21 patients (10.19%) developed postoperative infections. Serum IL-6, PCT, and CRP levels in the infected group were significantly higher than those in the non-infected group on PODs 3 and 5. IL-6 with an optimal cutoff value of 84.00 pg/mL (AUC 0.84), PCT with an optimal cutoff value of 1.39 ng/mL (AUC 0.80), CRP with an optimal cutoff value of 150.00 mg/L (AUC 0.76) on POD 3 had superior diagnostic accuracy in predicting postoperative infections. Multivariate analysis identified PCT and IL-6 levels on POD 3 as independent risk factors, the AUC of the combination of IL-6 and PCT was 0.89. The Delong test showed no difference between the AUC of IL-6 alone and IL-6 combined with PCT prediction (*P* = 0.07, Z = 1.81).

**Conclusions:**

IL-6 level on POD 3 is an excellent predictor of infectious complications following laparoscopic gastric cancer surgery. Patients with IL-6 levels lower than 84.00 pg/mL on POD 3 can ensure safe early discharge with a low probability of infection.

## Introduction

Gastric cancer (GC), one of the most malignant tumors of the digestive system, ranks fifth in incidence and fourth in cancer-related mortality globally [1]. Currently, the standard treatment for locally advanced GC is surgery-based comprehensive treatment [2, 3]. Surgery has survival benefits but can also lead to complications. Postoperative infection is a common complication of GC surgery, which can not only prolong the hospital stays and increase the hospitalization cost but also be life-threatening in severe cases [4, 5]. Clinical practitioners typically observe clinical symptoms and signs of patients to diagnose infectious complications, which have a clear lag to detect infection. Therefore, it is particularly important to explore novel markers for early prediction of infectious complications following GC surgery.

Recently, numerous studies have shown that white blood cell (WBC) count, C-reactive protein (CRP), and procalcitonin (PCT) are used to predict postoperative infectious complications [6–8]. However, the value of PCT and CRP levels in predicting infectious complications remains controversial [9, 10]. Furthermore, studies have revealed that these indicators are susceptible to surgery factors. Therefore, it is crucial to identify predictive indications that are more sensitive and less susceptible to external intervention for the early detection of postoperative infections. Interleukin 6 (IL-6), a cytokine, peaks and returns to normal levels more quickly than CRP during the acute inflammatory response, suggesting that it is a more sensitive indicator of the inflammatory response. Moreover, IL-6 has been used to predict infectious diseases, such as surgical site infection, nosocomial infection, and post-operative lung infection [11–13]. Additionally, one study reported that IL-6 could be a diagnostic marker of anastomotic leaks following colorectal cancer surgery [14].

Currently, there have been no reports of IL-6 as a diagnostic indicator of infections after GC surgery. To further promote the development of enhanced recovery after surgery (ERAS) in laparoscopic gastric cancer (LGC) surgery, we evaluated and compared the predictive values of CRP, PCT, and IL-6 for infectious complications following LGC surgery.

## Materials and methods

### Study design

This study was a cohort study. The inclusion criteria were as follows: (1) age ≥ 18 years; (2) preoperative gastroscopic biopsy confirming the diagnosis of GC; (3) absence of distant metastases; and (4) complete resection. The exclusion criteria were as follows: (1) preoperative infection; (2) open surgery or conversion to open surgery (when laparoscopic surgery is difficult to perform and cannot continue, switch to open surgery); (3) emergency surgery; and (4) incomplete data. Patients undergoing LGC surgery at the Department of Gastrointestinal Surgery at Wuhan Union Hospital of Tongji Medical College of Huazhong University of Science and Technology from January 2020 to June 2022 were enrolled. The ethical committee of the Union Hospital of Tongji Medical College, Huazhong University of Science and Technology, approved the study design. The waiver for informed consent was granted. The ethical approval number of this research was [2023] lunshenzi (0450).

### Date collection

Data on age, sex, body mass index (BMI), diabetes, smoking, preoperative chemotherapy, abdominal surgery, American Society of Anesthesiology (ASA) score, type and duration of surgery, extent of the resection, tumor-lymph node-Metastasis (TNM) staging, serum IL-6, PCT, and CRP levels, postoperative hospital stay, and postoperative infection were retrospectively collected. The cancer stage was classified according to the eighth edition of the American Joint Committee on Cancer System for GC [15]. The postoperative infectious complications were categorized using the modified Clavien-Dindo classification [16]. IL-6, PCT, and CRP levels were assessed before surgery and on postoperative days (PODs) 3 and 5. Plain computed tomography (CT) assessments of the lungs, abdomen, and pelvis were routinely performed on the POD 5. Prophylactic antibiotics were used for 48 h after surgery. For non-infections, antibiotics will be discontinued on POD 3. For the infections group, antibiotics will continue to be used. If the infection is not controlled or worsened, higher-level antibiotics will be used.

### Diagnostic criteria for postoperative infection

Anastomotic leak, widespread peritoneal infection, and abdominal abscess are all considered as intra-abdominal infections. A diagnosis of intra-abdominal infection is made when any of the following conditions exist: (1) abdominal abscess (collection of pus confirmed by percutaneous drainage); (2) pancreatic fistula (drain output with an amylase content greater than three times the serum amylase level; and (3) anastomotic leakage (positive-contrast swallow test) [17]. Pulmonary infection is clinical signs of pneumonia with radiographic evidence and positive sputum culture or bronchoalveolar lavage. Wound infection (purulent exudate in the wound with positive bacterial culture) usually develops within 30 days of surgery [18]. All patients were followed up at least 30 days after surgery.

### Statistical methods

SPSS statistical software (version 25, SPSS Inc., Chicago, IL, USA) and GraphPad Prism 8v8.0.0 were used for statistical analysis. Categorical data were presented as percentages (%), and the chi-square test or Fisher’s test was used for comparison. The measurement data were expressed as mean ± SD, and the t-test was used to compare the groups. The receiver operating characteristic (ROC) curve was used to evaluate and compare the predictive value of IL-6, PCT, and CRP in postoperative infection after LGC. The area under the curve (AUC) was calculated along with sensitivity, specificity, Youden index, negative predictive value (NPV), positive predictive value (PPV), and best critical value. The AUCs of the two ROCs were compared using the DeLong test. Statistical significance was set at *P* < 0.05.

## Results

### Clinicopathological characteristics

**Fig. 1:**
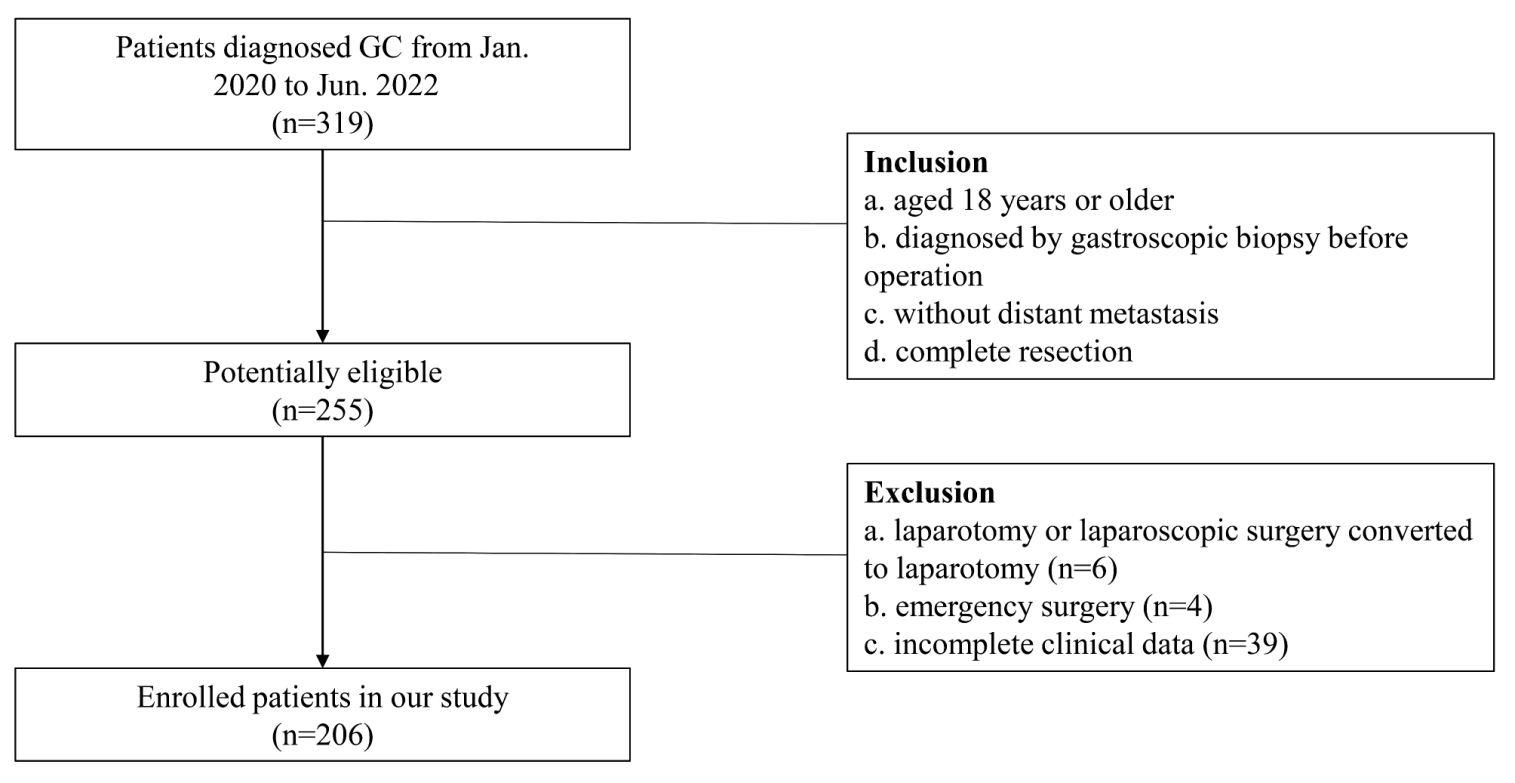
Flow chart illustrating the selection of objective patients

A total of 206 patients were enrolled in this study based on the inclusion and exclusion criteria (Fig. 1). Patients were divided into two groups depending on whether an infection occurred after surgery. Table 1 presents the patient characteristics of the two groups. There were 138 men and 68 women, with a mean age of 58.90 ± 10.50 years. One hundred and fourteen patients (55.40%) underwent distal gastrectomy, 19 (9.20%) proximal gastrectomy, and 73 (35.40%) total gastrectomy. The mean hospital stay following surgery was 10.00 ± 5.70 days.

**Table 1:** Clinicopathological characteristics of the entire study cohort

Clinical characteristics	Non-infections (n = 185)	Infections (n = 21)	*P* value
**Gender**			
Male	122	16	0.334
Female	63	5	
Age (y)	58.20 ± 10.30	64.80 ± 10.20	0.849
BMI (kg/m^2^)	22.87 ± 3.20	23.10 ± 3.00	0.738
**ASA**			
I	75	5	0.076
II	106	14	
III	4	2	
**Diabetes mellitus**			
Present	11	1	0.826
Absent	174	20	
**Smoking**			
Present	38	6	0.395
Absent	147	15	
**History of abdominal surgery**			
Present	38	5	0.727
Absent	147	16	
**Preoperative chemotherapy**			
Present	34	4	0.940
Absent	151	17	
**Extent of resection**			
Distal gastrectomy	101	13	0.699
Proximal gastrectomy	18	1	
Total gastrectomy	66	7	
Operation time(min)	292.50 ± 92.70	342.20 ± 109.80	0.215
**TNM stage**			
0	4	0	0.793
IA	53	4	
IB	17	3	
IIA	26	3	
IIB	17	3	
IIIA	25	5	
IIIB	22	2	
IIIC	12	1	
complete remission	9	0	
Postoperative hospital stays (d)	8.80 ± 2.50	21.40 ± 11.00	**< 0.001**

Age, BMI, operation time, and postoperative hospital stays are expressed as the mean ± SD

ASA = American Society of Anesthesiology, BMI = body mass index

Of the 206 patients, 21 developed infections (10.19%), including 12 with abdominal infections (5.83%), 8 with pneumonia (3.88%), and 1 with wound infection (0.48%). The median time to diagnosis of infection was 8 days (6–10 days).

### Comparison of serum IL-6, PCT, CRP and WBC levels between the two groups

**Fig. 2:**
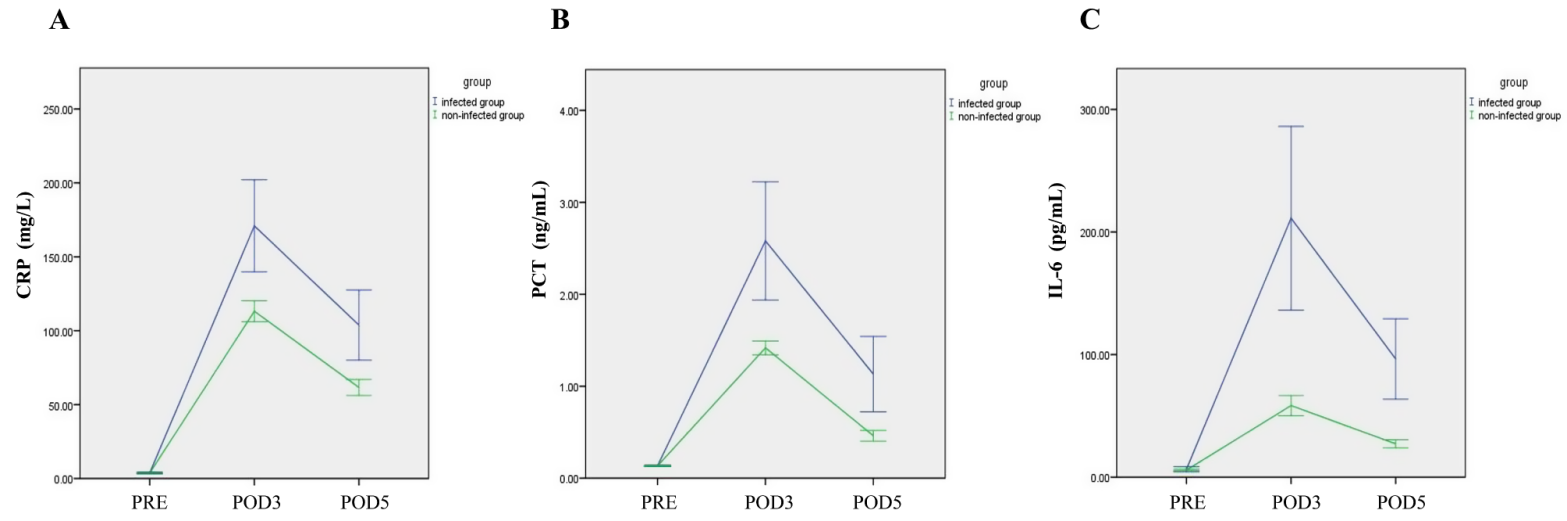
Preoperative and postoperative changes in CRP levels (**A**), PCT levels (**B**), and IL-6 levels (**C**) of patients with infection and non-infection

There was no difference between the two groups in the preoperative serum IL-6, PCT, CRP, and WBC levels (*P* > 0.05). Postoperative IL-6, PCT, CRP, and WBC values were higher in the infected group than in the non-infected group on PODs 3 and 5. On POD 3, the mean IL-6 levels in the infected group were 211.23 ± 164.63 pg/mL and the mean IL-6 levels in the non-infected group were 58.41 ± 56.84 pg/mL (*P* < 0.001) (Table 2). Postoperative serum IL-6, PCT, and CRP levels in the infected group and in the non-infected group peaked on POD 3 (Fig. 2).

**Table 2:** Comparison of daily levels in patients with and without infection

Date	Without Infection (n = 185)	With Infection (n = 21)	*P* Value
**PRE**			
WBC (G/L)	5.15 ± 1.15	5.68 ± 2.13	0.279
RBC (G/L)	4.00 ± 0.99	3.77 ± 0.61	0.317
PLT (G/L)	203.59 ± 68.48	205.90 ± 66.34	0.883
HB (g/L)	119.50 ± 21.30	114.19 ± 22.81	0.284
NEUT (G/L)	2.95 ± 1.24	3.51 ± 1.77	0.174
LY (G/L)	1.57 ± 0.60	1.51 ± 0.46	0.698
Alb (g/L)	38.00 ± 4.34	36.97 ± 3.42	0.291
D-D (mg/L)	0.25 ± 0.07	0.23 ± 0.06	0.411
CRP (mg/L)	3.70 ± 1.91	3.75 ± 1.47	0.917
PCT (ng/mL)	0.13 ± 0.03	0.13 ± 0.01	0.959
IL-6 (pg/mL)	5.78 ± 4.60	6.43 ± 4.57	0.539
**POD 3**			
WBC (G/L)	9.29 ± 3.17	12.23 ± 5.26	**0.020**
CRP (mg/L)	113.18 ± 48.39	170.96 ± 68.43	**< 0.001**
PCT (ng/mL)	1.42 ± 0.52	2.58 ± 1.41	**0.001**
IL-6 (pg/mL)	58.41 ± 56.84	211.23 ± 164.63	**< 0.001**
**POD 5**			
WBC (G/L)	6.84 ± 2.30	8.93 ± 3.40	**0.012**
CRP (mg/L)	61.61 ± 37.46	103.78 ± 52.04	**0.002**
PCT (ng/mL)	0.46 ± 0.41	1.13 ± 0.90	**0.003**
IL-6 (pg/mL)	27.21 ± 23.00	96.43 ± 72.09	**< 0.001**

PRE = preoperative, POD = Postoperative day, WBC = white blood cell, RBC = red blood cell, PLT = blood platelet, HB = hemoglobin, NEUT = neutrophil, LY = lymphocyte; Alb = albumin; D-D = D-dimer; CRP = C-reactive protein; PCT = procalcitonin; IL-6 = interleukin-6

Bold values indicate statistical significance at a *P* < 0.05

### Comparison of the accuracy of IL-6, PCT, and CRP levels in predicting postoperative infection

**Fig. 3:**
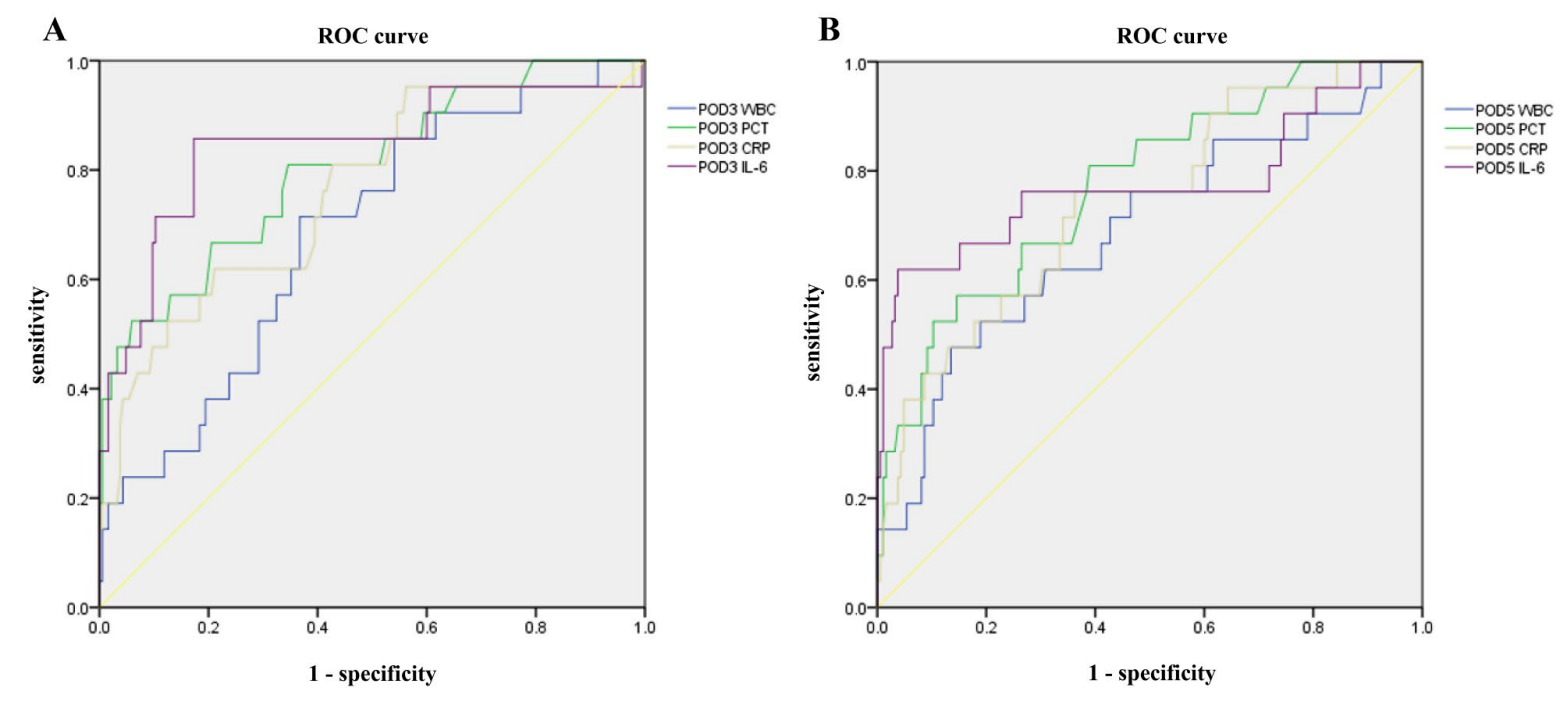
ROC curve analysis was performed to predict the predictive values of WBC, CRP, PCT, and IL-6 for postoperative infection on POD 3 (**A**) and POD 5 (**B**)

Based on the ROC curve, the accuracy of IL-6, PCT, CRP, and WBC levels on PODs 3 and 5 in predicting postoperative infection was calculated. The results are presented in Table 3; Fig. 3. IL-6 on POD 3 predicted infection with an AUC of 0.84, the cutoff value of 84.00 pg/mL, a sensitivity of 85.70%, and a specificity of 82.70%, PPV of 36.00%, and NPV of 98.10%.

**Table 3:** Comparison of the accuracy of CRP, PCT, and IL-6 to predict infection

Date	Cut-off value	AUC (95% CI)	Youden index	Sensitivity (%)	Specificity (%)	PPV (%)	NPV (%)	*P* Value
**POD 3**								
CRP (mg/L)	150.00	0.76	0.41	61.90	78.90	24.50	94.80	**< 0.001**
PCT (ng/mL)	1.39	0.80	0.46	81.00	65.40	21.00	96.80	**< 0.001**
IL-6 (pg/mL)	84.00	0.84	0.68	85.70	82.70	36.00	98.10	**< 0.001**
**POD 5**								
CRP (mg/L)	64.50	0.74	0.40	76.20	63.80	19.30	95.90	**< 0.001**
PCT (ng/mL)	0.77	0.77	0.43	57.10	85.40	30.80	94.60	**< 0.001**
IL-6 (pg/mL)	58.10	0.78	0.58	61.90	96.20	41.90	95.40	**< 0.001**

CRP = C-reactive protein, PCT = procalcitonin, IL-6 = interleukin-6, POD = Postoperative day, AUC = area under curve, PPV = positive predictive value, NPV = negative predictive value

Bold values indicate statistical significance at a *P* < 0.05

### Correlation analysis of IL-6 elevation and length of hospital stay

In the infected group, the mean postoperative hospital stay was 21.40 ± 11.00 days, whereas in the non-infected group, it was 8.80 ± 2.50 days (*P* < 0.001). Furthermore, we used the cut-off value of 84.00 pg/mL for IL-6 on POD3 to divide the samples into two groups, and analyze the differences in hospital stay based on this cut-off value. The mean hospital stays of the two groups were 9.20 ± 4.00 days and 12.90 ± 8.70 days, respectively (*P* < 0.001).

### Predictors of postoperative infection

Based on these findings, we discovered that the prediction of postoperative infection on POD 3 was more accurate than that on POD 5. To predict postoperative infection in LGC, we performed univariate and multivariate analyses of IL-6, PCT, and CRP levels, and WBC on POD 3. PCT level > 1.39 ng/mL (odds ratio [OR]: 4.23, 95% confidence interval [CI]: 1.15–15.63) and IL-6 level > 84.00 pg/mL (OR: 21.81, 95% CI: 5.68–83.76) on POD 3 were independent predictors of postoperative infection in LGC (Table 4).

**Table 4:** Univariate and multivariate analyses of factors for postoperative infection

Clinical characteristics	Univariate analysis		*P* Value	Multivariate analysis		*P* Value
	Total patients	Patients with infection		OR	95% CI	
**WBC on POD 3 (G/L)**						
≤9.81	123	6	**0.002**			
>9.81	83	15				
**CRP on POD 3 (mg/L)**						
≤150	154	8	**< 0.001**			
>150	52	13				
**PCT on POD 3 (ng/mL)**						
≤1.39	125	5	**< 0.001**	4.23	(1.15, 15.63)	**0.030**
>1.39	81	16				
**IL-6 on POD 3 (pg/mL)**						
≤84	156	4	**< 0.001**	21.82	(5.68, 83.76)	**< 0.001**
>84	50	17				

POD = Postoperative day, CRP = C-reactive protein, PCT = procalcitonin, IL-6 = interleukin-6, OR = odds ratio, CI = confidence interval

Bold values indicate statistical significance at a *P* < 0.05

### Accuracy of IL-6 combined with PCT in predicting postoperative infection

**Fig. 4:**
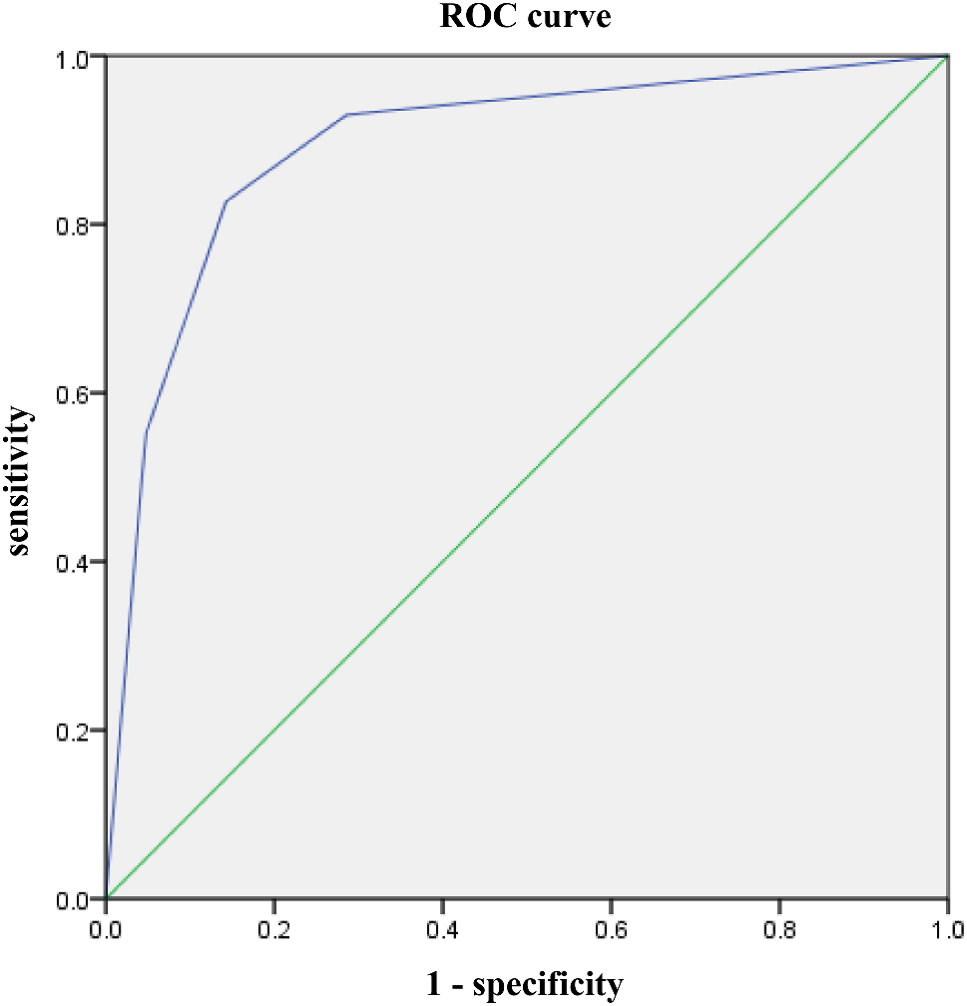
ROC curve analysis for IL-6 combined PCT in predicting postoperative infection on POD 3

To further improve the accuracy of predicting infectious complications after LGC, we combined IL-6 with PCT to predict infection; the results are shown in Table 5; Fig. 4. We found that the AUC of IL-6 combined with PCT for predicting postoperative infection was 0.89 (95% CI: 0.84–0.93). Using the DeLong test, there was no significant difference between the AUC of IL-6 combined with PCT on POD 3 and the AUC of IL-6 alone on POD 3 (*P* = 0.07, Z = 1.81).

**Table 5:** Comparison of ACU between IL-6 alone and IL-6 combined with PCT in predicting infection

	AUC	95% CI	Z Value	*P* Value
IL-6 alone	0.84	(0.79, 0.89)	1.81	0.07
IL-6 combined with PCT	0.89	(0.84, 0.93)		

PCT = procalcitonin, IL-6 = interleukin-6, AUC = area under curve

Bold values indicate statistical significance at a *P* < 0.05

## Discussion

In this study, we found for the first time that IL-6 levels on POD 3 could be used for the early predictor of postoperative infectious complications in LGC, and its predictive value was better than that of PCT and CRP. Multivariate analysis showed that elevated IL-6 and PCT levels on POD 3 were independent factors for postoperative infection complications. Furthermore, there was no significant difference in the predictive value between PCT combined with IL-6 and IL-6 alone on POD 3, indicating that IL-6 alone is a good predictor of postoperative infection.

The CLASS01 study revealed that laparoscopic minimally invasive surgery is surgical alternatives for patients with advanced GC [19]. In recent years, laparoscopic procedures have gained popularity in the field of GC surgery. Despite the progress of minimally invasive surgery technology and the strengthening of perioperative nursing, there is still a certain incidence of infectious complications after surgery. Studies have reported that the incidence of abdominal infection complications after GC surgery was 3.30–10.60% [20]. In the present study, the rate of infectious complications following LGC surgery was 10.19%, with abdominal infections accounting for 5.83%, and pneumonia accounting for 3.80%. The infections pose a significant barrier to the rapid recovery from GC after surgery. Therefore, early detection and treatment should be achieved to reduce the risk of infectious complications after LGC surgery.

Recently, WBC, CRP, and PCT have been widely used to diagnose postoperative infection problems in clinical practice. CRP is an acute temporal protein that is synthesized by hepatocytes in response to IL-6 stimulation. According to a previous study, serum CRP on PODs 3 or 4 can be utilized as an early indicator of infectious postoperative complications [21, 22]. In the present study, we discovered that CRP levels were considerably higher in the infected group than in the non-infected group on PODs 3 and 5. When CRP was used to predict infection at a critical value of 150.00 mg/L on POD 3, the sensitivity and specificity were 61.90% and 78.90%, respectively. Therefore, the CRP level on POD 3 has favorable clinical guideline relevance for predicting postoperative infection. PCT is synthesized mainly by thyroid C cells. When tissues are stimulated by bacterial endotoxins, the blood PCT levels rapidly increased. Therefore, PCT can be used as an indicator of severe inflammation, infection, and sepsis. An increasing number of studies have shown that PCT can be used as a sensitive indicator for the early prediction of postoperative infection after surgery [6, 23–25]. Tatsuoka et al. [26] reported that serum PCT level on POD 4 was effective in the early detection of postoperative infection after laparoscopic colorectal resection. Sponholz et al. [25] reported that PCT was useful for detecting infectious complications after heart surgery.

In comparison, there are few studies on the use of PCT for predicting infections after GC surgery. Xiao et al. [27] found that PCT is a potential indicator of postoperative infectious complications in patients with GC. They revealed that postoperative infections were less likely to occur when PCT levels on POD 3 < 0.70 ng/mL. However, both open and laparoscopic surgeries were included in their study, and only PCT and WBC were analyzed, while CRP was not included. In addition, there has been controversy regarding the value of CRP and PCT in predicting postoperative infectious complications in gastrointestinal tumors. Therefore, we investigated the predictive values of PCT and CRP levels for postoperative infectious complications in LGC. We found PCT had a greater AUC and NPV than CRP when PCT reached a critical threshold of 1.39 ng/mL on POD 3, indicating that PCT may be a stronger predictor of infection following LGC than CRP.

IL-6, a 185-amino acid polypeptide, can be detected in the serum during early infection. Serum IL-6 is markedly increased in response to bacterial infection, activating monocytes and macrophages and inducing the release of CRP and PCT [28, 29]. IL-6 may be helpful in the earlier prediction of infection to some extent because it is elevated in infection before PCT and CRP. A meta-analysis showed that serum IL-6 has moderate diagnostic value and potential clinical value in differentiating infection in critically ill patients [30]. Chen et al. [13] showed that IL-6 on PODs 1 and 2 had the highest diagnostic value in patients with complications of pneumonia after cardiac surgery, with AUCs of 0.78 and 0.77, respectively; thus, IL-6 was more sensitive for the diagnosis of early postoperative pneumonia after cardiac surgery. Lensk et al. [11] reported the high value of IL-6 for the diagnosis of spinal surgical site infection, with IL-6 > 15.30 pg/mL and an AUC of 0.95 indicating surgical site infection. Rettig et al. [31] showed that elevated IL-6 levels on POD 1 were associated with postoperative complications in major abdominal surgery, with a predictive complication AUC of 0.67, whereas CRP only had value in differentiating complications on POD 3. All these studies demonstrated that IL-6 has good utility in predicting postoperative infectious complications.

In recent years, researchers have shown that IL-6 has a high predictive value for infectious complications following gastrointestinal surgery. Montero et al. [32] reported that serum IL-6 performed better in distinguishing complicated from simple pediatric acute appendicitis, with a median IL-6 level of 17.20 pg/mL in uncomplicated patients and 60.26 pg/mL in complicated patients. Xie et al. [33] found that high postoperative IL-6 levels were associated with the development of postoperative infectious abdominal complications in patients undergoing elective surgery for Crohn’s disease. Jerome et al. [34] reported that IL-6 had a higher diagnostic accuracy than that of PCT in predicting infection after major gastrointestinal surgery. After reviewing relevant literature (Table 6), we found no any studies on IL-6 predicting postoperative infection in GC. We investigated the utility of IL-6 in predicting infectious problems following LGC surgery. According to our findings, postoperative serum IL-6 levels in the infected group were considerably greater than those in the non-infected group, and the predictive value on POD 3 was higher than that on POD 5.

**Table 6:** Summary of studies on IL-6 in predicting complications after surgery

References	Study design	Study interval	Object	N	
Lenski et al. [11]	Prospective	2011–2016	Spine surgery	98	
Song et al. [7]	Retrospective	2017–2018	General surgery	3810	
Yu et al. [12]	Prospective	2017	General surgery	146	
Montero et al. [32]	Prospective	2021	Pediatric acute appendicitis (PAA)	205	
Chen et al. [13]	Prospective	2020–2021	Cardiac surgery	694	
Rettig et al. [31]	Prospective	Without introduction	Major abdominal surgery	137	
Xie et al. [33]	Prospective	2014–2016	Crohn’s disease	118	
Endpoint		Time point	Cut-off value or AUC of IL-6		
Surgical site infection		The day of diagnosis of SSI	15.30 pg/mL		
Intra-abdominal infection		POD 1 and POD 3	0.79 on POD 3		
Nosocomial infection		POD 1 and POD 2	0.86 on POD 1		
Complicated PAA		Prior to the scheduled intervention	60.25 pg/mL		
Postoperative pneumonia		PODs 1, 2, 3, 4, and 5	0.78 on POD 1		
Postoperative complications		PODs 0, 1, 3, and 7	432.00 pg/mL on POD 1		
Intra-abdominal septic complications		PODs 0, 1, 3, and 5	0.71 on POD 1		

Additionally, IL-6 > 84.00 pg/mL and PCT > 1.39 ng/mL on POD 3 were found to be independent predictors when we analyzed the predictors of postoperative infection. To further improve the accuracy of predicting postoperative infection, we analyzed the value of PCT combined with IL-6 in predicting postoperative infection. The results showed that the AUC of IL-6 combined with PCT was 0.89. Furthermore, we compared the values of IL-6 alone and PCT combined with IL-6 in predicting postoperative infection on POD 3 after GC surgery. The results showed no significant difference between the two groups. Therefore, IL-6 alone is an excellent predictor of postoperative infection without the need for combined PCT and CRP levels, eliminating the needless testing and saving the hospital expenses.

This study had some limitations. First, its retrospective design and small sample size make it vulnerable to bias. Additionally, this study only examined the overall postoperative infectious complications. Therefore, we intend to use a larger sample size to further investigate the role of IL-6 in various infections following LGC surgery.

## Conclusion

IL-6 on POD 3 is an excellent negative predictor of infectious complications following LGC surgery. Compared with CRP and PCT, IL-6 has a higher predictive value for postoperative infections. Patients with IL-6 levels lower than 84.00 pg/mL on POD 3 can ensure safe early discharge with a low probability of infections.

## Data Availability

The data used to support the findings of this study are available from the corresponding authors upon request.

## Abbreviations

CRP: C-reactive protein
PCT: procalcitonin
IL-6: interleukin-6
POD: postoperative day
AUC: area under the curve
PPV: positive predictive value
NPV: negative predictive value
GC: gastric cancer
WBC: white blood cell
ERAS: enhanced recovery after surgery
LGC: laparoscopic gastric cancer
BMI: body mass index
ASA: American Society of Anesthesiology
TNM: tumor-lymph node-Metastasis
CT: computed tomography
ROC: receiver operating characteristic
OR: odds ratio
